# Few-shot skin lesion classification with Adaptive Multi-Scale Convolutional Attention Network

**DOI:** 10.1371/journal.pone.0351318

**Published:** 2026-06-25

**Authors:** HuiYing Jin, E. Liu, Qin Xu, YuChen Li, XiaoLiang Chen, WeiHua Chen

**Affiliations:** 1 Department of Laboratory Medicine, Hospital of Chengdu University of Traditional Chinese Medicine, Chengdu, China; 2 Chengdu University of Traditional Chinese Medicine, Chengdu, China; 3 Anesthesiology Department/Operating Room, Hospital of Chengdu University of Traditional Chinese Medicine, Chengdu, China; 4 College of Electronic and Information Engineering, Tongji University, Shanghai, China; The University of Texas, MD Anderson Cancer Center, UNITED STATES OF AMERICA

## Abstract

Computer-aided diagnosis of skin lesions faces core challenges, including scale diversity, blurred boundaries, intra-class morphological variations, and sparse data. Existing methods often rely on fixed receptive fields or generic attention mechanisms, struggling to fully adapt to the unique characteristics of skin lesions. To address this, we propose an Adaptive Multi-scale Convolutional Attention Network (AMCANet), which aims to achieve accurate and robust classification of skin lesions with limited data. AMCANet comprises three core modules: the adaptive multi-scale convolution module dynamically adjusts the receptive field to accommodate lesions of varying sizes; the hierarchical channel attention module integrates multi-level semantic information across different resolutions; and the skin spatial attention module leverages image gradient information to enhance lesion boundaries and local texture features. Extensive few-shot experiments on the HAM10000 and PAD-UFES-20 public datasets demonstrate that AMCANet significantly outperforms existing baseline models across multiple metrics, exhibiting promising generalization capabilities on the evaluated datasets. Qualitative and visual analyses further validate the model’s ability to extract discriminative features and effectively focus on lesion regions. This study proposes a deep learning model, which demonstrates certain effectiveness in classifying skin lesions even with a small number of samples, providing a potential direction for future research.

## Introduction

According to the World Health Organization, more than 3 million new cases of skin cancer are diagnosed globally every year. Among these, melanoma, due to its high metastatic potential, is the deadliest subtype. Early diagnosis can increase the 5-year survival rate of melanoma patients from less than 20% (in advanced stages) to over 99% (in early stages, with tumor thickness ≤ 1.0 mm) (For reports on skin cancer, please refer to: https://seer.cancer.gov/). This significant difference highlights the critical importance of timely and accurate diagnosis in improving patient prognosis. Therefore, developing high-performance medical image analysis systems has become a key focus in the global healthcare sector. Deep learning [1,2] (DL) technology has demonstrated remarkable capabilities in extracting discriminative features from complex medical images. Among these, Convolutional Neural Networks (CNNs) have achieved outstanding results in image recognition [3], with applications spanning across various imaging modalities such as Computed Tomography (CT) scans [4] and Magnetic Resonance Imaging (MRI) [5]. DL-based computer-aided diagnostic systems have become powerful tools in addressing the challenges faced in modern healthcare.

In clinical practice, dermoscopy has become an indispensable tool for diagnosing skin lesions [6,7]. It can clearly present subcutaneous structures that are not visible to the naked eye, such as pigment networks, capillary patterns, and keratin deposits. However, interpreting dermoscopic images requires extensive professional training and clinical experience. The subjectivity and variability of this visual diagnosis are particularly pronounced in areas with limited medical resources, resulting in diagnostic delays and increased patient mortality. The limitations of manual diagnosis underscore the need for automated skin lesion classification systems [8,9], which must deliver consistent and accurate assessment results across diverse clinical scenarios. Using artificial intelligence [10,11] (AI) algorithms to analyze dermoscopic images can assist clinicians in early detection and precise classification of skin lesions [12,13], thereby reducing diagnostic errors. AI technology has shown tremendous potential in this field, with several deep learning models achieving significant success in classifying common skin lesions (e.g., melanoma, basal cell carcinoma). For example, deep learning models like HybridConvNeXt [14], MATNet [15], and MuRANet [16] have achieved test accuracies over 91% on the HAM10000 dataset [17], fully validating the feasibility of data-driven AI for skin lesion classification.

Despite significant progress, skin lesion classification [14–16] still faces four core challenges. First, there is a vast difference in lesion size, ranging from tiny spots of 2–3 mm to large nodules or plaques over 10 cm in diameter [18]. Traditional single-scale convolutional networks, which use a fixed receptive field, struggle to simultaneously capture the details of small lesions and the global structure of large lesions. Second, boundary ambiguity is commonly seen in early or morphologically irregular lesions, where the transition zone between lesion tissue and normal skin tissue lacks a clear demarcation [19]. Third, factors such as skin type, lesion location, and disease stage cause significant morphological differences within the same skin lesion category [20]. For instance, melanoma can appear as a flat macule, raised nodule, or ulcerated plaque, each varying in color and texture. Fourth, the few-shot problem arises from the scarcity of labeled medical data [21], especially for rare lesion subtypes. For example, in the PAD-UFES-20 dataset [22], the total samples for common lesions like melanoma are only 52, while squamous cell carcinoma has just 192 samples. The limited number of samples can easily lead to model overfitting and poor generalization.

Existing skin lesion classification models [13–16,23] still have many limitations in addressing the challenges mentioned above, resulting in suboptimal performance. Most methods rely on simple stacking of fixed-size convolutional kernels to extract multi-scale features. This rigid design lacks adaptability to the diverse size distribution of skin lesions and often introduces irrelevant contextual information, weakening the discriminative power of key features. Additionally, current models primarily use generic feature extraction frameworks developed for natural images, without tailoring them for the unique characteristics of skin lesion images (e.g., specific textures). Third, existing attention models mainly focus on global semantic information, rather than the local structural details and boundary features needed for skin lesion analysis. Consequently, these generic attention models are less sensitive to the subtle differences between skin lesions and normal tissue.

To address these challenges and limitations, we propose an Adaptive Multi-Scale Convolutional Attention Network (AMCANet), which integrates three specialized modules to achieve robust and accurate few-shot skin lesion classification. The proposed framework consists of three core components: the Adaptive Multi-Scale Convolutional Module, the Hierarchical Channel Attention Module, and the Skin-Space Attention Module. Specifically, AMCANet first utilizes the Adaptive Multi-Scale Convolutional Module to dynamically adjust the receptive field based on lesion size, enhancing its ability to handle multi-size lesions. Then, the Hierarchical Channel Attention Module analyzes the importance of channels at different resolutions and integrates multi-level semantic information. Next, the Skin-Space Attention Module uses image gradient information to highlight lesion boundaries and strengthen skin-specific features. Together, these components achieve adaptive feature extraction, skin feature enhancement, and hierarchical fusion, providing a robust foundation for classification. We conducted extensive experiments and analyses on two public datasets under few-shot settings, and the results show that the proposed AMCANet achieves the highest performance in all few-shot scenarios.

The main contributions of this paper are summarized as follows:

- We propose a novel Adaptive Multi-Scale Convolutional Attention Network (AMCANet), which effectively mitigates key challenges in skin lesion classification, such as scale diversity, boundary ambiguity, and intra-class morphological differences through three specially designed modules.
- We design a multi-scale adaptive feature extraction mechanism tailored for the characteristics of skin lesion images. By dynamically adjusting the convolutional receptive field, the model can adaptively fuse multi-scale features based on lesion size, avoiding the introduction of irrelevant contextual information, and enhancing its ability to represent lesions of various sizes.
- We integrate hierarchical channel attention with gradient-guided spatial attention mechanisms, enhancing the model’s sensitivity to local structure and texture in skin lesions, especially under few-shot conditions, demonstrating strong generalization performance.
- We conduct comprehensive few-shot experiments on several public datasets, and the results show that AMCANet significantly outperforms existing advanced models in key metrics like accuracy and F1 score. Additionally, through confusion matrix analysis, feature visualization, and attention heatmap interpretation, we further verify the superiority of the model, offering a potential technical approach for further exploration in few-shot skin lesion auxiliary diagnosis.

## Related works

### Skin lesion classification

Skin lesion classification has made significant progress in recent years through deep learning. Existing research primarily focuses on directions such as feature extraction optimization, knowledge transfer efficiency, enhancement of attention mechanisms, and innovation in supervision strategies. In the field of knowledge distillation, Yu et al. [24] proposed a Variational AdaBoost Knowledge Distillation framework, which breaks the traditional passive imitation of teacher knowledge by student models. Through AdaBoost, the student model actively mines teacher knowledge granularity based on its own learning difficulties and introduces a variational difficulty mining strategy. It uses graph convolution networks to capture multi-hop neighbor information and maximizes teacher-student mutual information to filter out noise interference. In terms of lightweight model design, Sulthana et al. [25] proposed the S-MobileNet architecture, which improves image preprocessing by optimizing the Gaussian filtering algorithm and segmentation-based fractal feature extraction. They also use the Mish activation function to alleviate the neuron death issue of ReLU and apply L1 norm pruning to intermediate layers, balancing both lightweight design and diagnostic performance. Aruk et al. [14] combined the advantages of ConvNeXt and Vision Transformer (ViT), using ConvNeXt to extract fine-grained features such as local texture and edges, while the Transformer captures long-range dependencies within lesion regions, effectively mitigating the class imbalance problem. In terms of innovation in attention mechanisms, Roy et al. [23] proposed a Background-Invariant Independent Guided Multi-Head Attention Network, which guarantees the independence of neurons between attention heads using the Hilbert-Schmidt Independence Criterion, reducing feature redundancy. They also incorporate a saliency-driven background vector shuffling mechanism, enhancing the model’s robustness to background noise. Gajera et al. [15] proposed MTA-Net, which designs a multi-scale triple attention module, including multi-scale triple spatial attention and channel attention. It uses dilated convolutions with varying dilation rates to capture multi-scale features, modeling fine-grained dependencies in both spatial and channel dimensions. Regarding supervision strategy optimization, Dzieniszewska et al. [26] proposed two deep pixel-level supervision methods, based on constant maps and segmentation masks, respectively. These methods provide supervision signals for each pixel in the feature map, guiding the network to focus on lesion areas and reducing background interference.

Although significant progress has been made in skin lesion classification, current research still lacks specialized designs addressing the scale diversity and boundary ambiguity of skin lesions. To address these limitations, we propose an Adaptive Multi-Scale Convolutional Attention Network to handle these challenges. Our approach dynamically adjusts the receptive field through multi-scale convolutions to adapt to lesions of different sizes. Then, it analyzes the importance of image channels at different resolutions to capture multi-level semantics, and finally combines image gradient information to emphasize lesion boundaries. The proposed method, through these steps, effectively addresses the limitations mentioned.

## Preliminaries

In this section, we formally define the few-shot skin lesion classification task and elaborate on the dataset splitting strategy employed in this paper. Let $D=\{(x_{i},y_{i}){\}}_{i=1}^{M}$ represent a dermoscopic image dataset consisting of *C* distinct classes, where *x*_*i*_ denotes the *i*-th skin lesion image, $y_{i}\in \{1,2,\ldots ,C\}$ corresponds to its categorical label, and *M* signifies the total number of samples within the dataset. Suppose the *c* -th class contains *M*_*c*_ samples, satisfying the constraint $\sum_{c=1}^{C}M_{c}=M$. Under the few-shot learning paradigm, we adhere to the experimental setup proposed in [27], which fixes the number of training samples per class as *K*. Concretely, for each class *c*, we randomly select *K* samples to construct the class-specific training subset $S_{\text{train}}^{c}$, randomly extract *V* samples to form the class-specific validation subset $S_{\text{val}}^{c}$, and all residual samples constitute the class-specific test subset $S_{\text{test}}^{c}$. The global training, validation, and test sets of the entire dataset are formulated as follows:


$$\begin{array}{c} S_{\text{train}}=\overset{C}{\underset{c=1}{⋃}}S_{\text{train}}^{c},\;S_{\text{val}}=\overset{C}{\underset{c=1}{⋃}}S_{\text{val}}^{c},\;S_{\text{test}}=\overset{C}{\underset{c=1}{⋃}}S_{\text{test}}^{c} \end{array}$$ (1)

where the three subsets are mutually exclusive, namely $S_{\text{train}}\cap S_{\text{val}}=\emptyset$, $S_{\text{train}}\cap S_{\text{test}}=\emptyset$, and $S_{\text{val}}\cap S_{\text{test}}=\emptyset$. The data partitioning scheme adopted in this study deviates substantially from the prevalent settings employed in conventional meta-learning-based few-shot learning paradigms. Typical meta-learning approaches, such as Prototypical Networks [28] and Model-Agnostic Meta-Learning (MAML) [29], generally adhere to the *N*-way *K*-shot task construction framework for few-shot learning. During the training phase, each training episode entails randomly sampling N distinct classes from the training dataset, followed by extracting K labeled samples per class to formulate the support set and *Q* samples per class to construct the query set, thereby generating an independent few-shot learning task. The model is trained across a multitude of such episodic tasks to acquire the capability of learning-to-learn, enabling rapid adaptation to novel, unseen tasks. Critically, the classes contained in the training set and test set are strictly mutually exclusive, which is designed to simulate the real-world scenario where the model encounters entirely novel classes during inference. In the testing stage, the model is further fine-tuned with a limited number of labeled samples (i.e., the support set) from new classes, and its classification performance is subsequently evaluated on the corresponding query set. In stark contrast to meta-learning methodologies, this work follows the standard few-shot learning protocol [27,30,31], where each class is assigned a fixed quantity of K training samples (e.g., 10-shot, 20-shot settings). The entire dataset is split into mutually exclusive training, validation, and test subsets. Specifically, the training set incorporates a small number of representative samples from all classes, while the validation and test sets consist of the remaining samples of the same classes with zero overlap across subsets. The model is trained directly on the fixed limited-scale training set via supervised learning to optimize the classification head, eliminating the need to construct a large number of dummy episodic tasks throughout the training procedure. At test time, the model performs direct classification on unseen test samples, without relying on extra auxiliary labeled samples for rapid task adaptation.

## Methods

**Fig 1:**
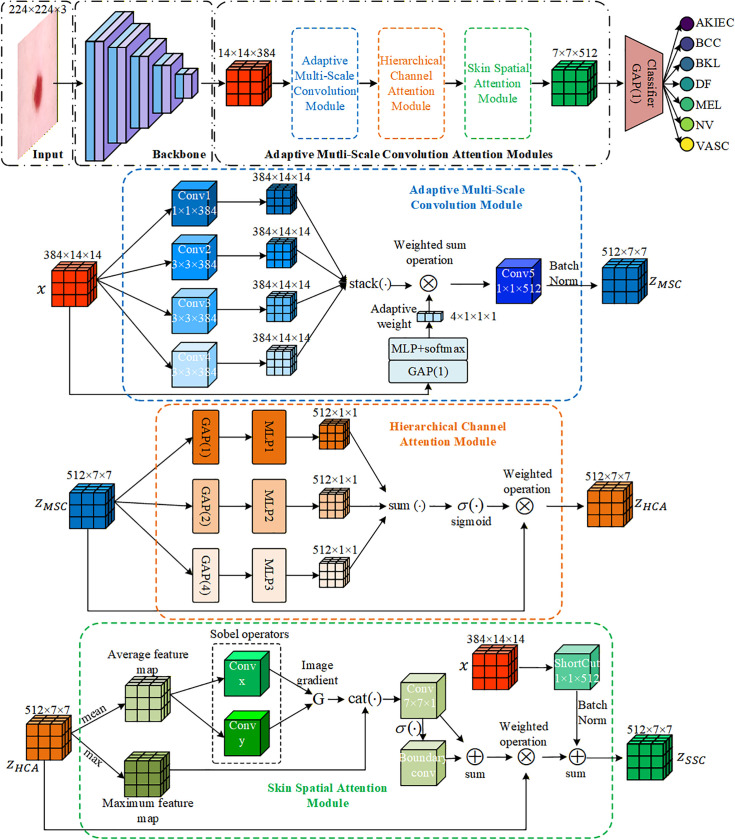
Overall architecture. The most important parts of AMCANet consist of an adaptive multi-scale convolution module, a hierarchical channel attention module, and a skin spatial attention module. Where the number is the size of the convolution kernel.

We propose the Adaptive Multi-Scale Convolutional Attention Network (AMCANet) (Our code is available from: https://github.com/11124asda/AMCANet) to address core challenges in skin lesion classification, such as scale diversity, boundary ambiguity, intra-class morphological differences, and limited sample data. AMCANet achieves adaptive feature extraction, multi-level semantic fusion, and skin-specific feature enhancement through three key innovative modules. The overall architecture is shown in Fig 1. The model first uses a backbone network to extract basic features, then passes through the Adaptive Multi-Scale Convolutional Module, Hierarchical Channel Attention Module, and Skin Spatial Attention Module sequentially. Finally, classification is performed through global average pooling and a fully connected layer. Below, we will detail the design motivations and structures of each module.

### Adaptive Multi-Scale Convolution module

Skin lesions exhibit significant size variations (ranging from small 2–3 mm early lesions to large patches exceeding 10 cm), making it difficult for traditional convolutional networks with fixed receptive fields to simultaneously balance global structure and local details. The Adaptive Multi-Scale Convolution (AMSC) module dynamically merges multi-scale features using adaptive weights, allowing the network to select the most suitable receptive field based on the size characteristics of the input lesion, avoiding irrelevant contextual information. The AMSC module consists of four parallel convolution branches, each using different convolution kernels and dilation rates to achieve multi-scale receptive fields. The multi-scale features are then weighted and fused through global average pooling and a multilayer perceptron (MLP).

Specifically, for a minibatch of skin lesion data $D\in {\mathbb{R}}^{B\times C\times H\times W}$, where *B*, *C*, *H* and *W* represent batch size, number of channels, image height, and width, we first extract the basic features of the image using the backbone, $x=\text{backbone}(D)\in {\mathbb{R}}^{B\times 384\times 7\times 7}$. Following the work of Aruk et al. [14], we use ConvNeXt-Tiny [32] as the backbone. The four-scale convolution results are:


$$\begin{array}{c} F_{k}={\text{Conv2d}}_{k}(x),k=1,2,3,4 \end{array}$$ (2)

The adaptive weights are obtained through the following process:


$$\begin{array}{c} w=\text{Softmax}(W_{2}\cdot \text{ReLU}(W_{1}\cdot \text{GAP}(x)+b_{1})+b_{2})\in {\mathbb{R}}^{B\times 4} \end{array}$$ (3)

where *W*_1_, *W*_2_, *b*_1_, and *b*_2_ represent the weights and biases of the fully connected layers in the multilayer perceptron. Finally, we fuse the features using weighted summation and apply a convolution to obtain the multi-scale features *z*_AMSC_:


$$\begin{array}{c} z_{\text{AMSC}}=\text{Conv2d}(\sum_{k=1}^{4}w_{k}⊗F_{k})\in {\mathbb{R}}^{B\times 512\times 7\times 7},k=1,2,3,4 \end{array}$$ (4)

where ⊗ denotes element-wise multiplication. In a few-shot learning scenario, for rare or few-sample lesion categories, the AMSC module can adjust the receptive field according to the inherent size characteristics of the lesion. This ability stems from the module’s parameter-sharing mechanism, where all samples share the same set of multi-scale convolution kernels but are fused using sample-specific weights, enabling generalization to rare lesion sizes.

### Hierarchical Channel Attention module

Traditional channel attention mechanisms rely only on the single-scale statistical information obtained from global average pooling (GAP), which is insufficient when dealing with skin lesion images that have complex hierarchical structures. Skin lesion features often exhibit multi-level semantic characteristics, with shallow features containing low-level visual cues such as edges and textures, while deep features encode high-level semantic information about the lesion category. The Hierarchical Channel Attention (HCA) module builds a multi-resolution feature pyramid to analyze channel importance at different spatial scales, thus achieving fusion and complementarity of multi-level semantic information.

Specifically, the HCA module contains three parallel pooling layers that downsample the feature map to sizes 1 × 1, 2 × 2, and 4 × 4, respectively. These are then mapped to channel weights through three multilayer perceptrons (MLPs). The three weights are averaged and activated using the Sigmoid function, resulting in the hierarchical fused channel attention map. This hierarchical design allows the network to simultaneously consider the global appearance, regional distribution, and local details of the lesion, creating a more comprehensive feature representation. The HCA module first accepts the output from the AMSC layer, then performs parallel pooling operations:


$$\begin{array}{c} P_{l}={\text{GAP}}_{S_{l}\times S_{l}}(z_{\text{AMSC}})\in {\mathbb{R}}^{B\times 512\times S_{l}\times S_{l}},l=1,2,3 \end{array}$$ (5)

where $S_{l}=1,2,4$. Next, we flatten *P*_*l*_ and obtain attention weights through three parallel MLPs:


$$\begin{array}{c} A_{l}=W_{4l}\cdot \text{ReLU}(W_{3l}\cdot \text{Flatten}(P_{l})+b_{3l})+b_{4l}\in {\mathbb{R}}^{B\times 512} \end{array}$$ (6)

where *W*_3*l*_, *W*_4*l*_, *b*_3*l*_ and *b*_4*l*_ represent the weights and biases of the fully connected layers of the three MLPs (*l* = 1, 2, 3). Finally, we fuse the weights and activate them, then apply them to the features to obtain the HCA feature representation output *z*_HCA_:


$$\begin{array}{c} Z_{\text{HCA}}=Z_{\text{AMSC}}⊗\sigma \left(\frac{1}{3}\sum_{i=1}^{3}A_{i}\right)\in {\mathbb{R}}^{B\times 512\times 7\times 7} \end{array}$$ (7)

In few-shot scenarios, the model tends to overly focus on feature patterns that appear by chance in the training set. The HCA module forces the model to consider features at different levels of abstraction by analyzing channel importance across multiple resolutions, increasing feature diversity. Furthermore, even if some channels are not important from a global pooling perspective, they may contain crucial discriminative information at local resolutions. This multi-level analysis ensures that such discriminative information is not neglected.

### Skin Spatial Attention module

In skin lesion diagnosis, the boundary features of lesions are of crucial clinical importance. Malignant lesions (such as melanoma) often exhibit irregular and blurred boundaries, while benign lesions typically have clear and regular boundaries. However, general spatial attention mechanisms tend to be insensitive to these medical-specific features. The innovation of the Skin Spatial Attention (SSA) module lies in combining image gradient information (reflecting boundary sharpness) with texture features (reflecting tissue heterogeneity), specifically optimizing the spatial features of skin lesion images.

Specifically, the SSA module first computes the average and maximum features of the input feature map, and uses the Sobel operator to calculate the gradient magnitude of the average feature as an edge hint. The gradient map is then concatenated with the maximum feature map and passed through a convolution layer to generate an initial attention map. Further refinement of the attention map is achieved using a lightweight boundary enhancement convolution network that highlights the transition regions of the lesion. The average and maximum features are calculated as follows:


$$\begin{array}{c} M_{\text{avg}}=\frac{1}{C}\sum_{i=1}^{C}z_{{\text{HCA}}_{:,i,:,:}},\;M_{\text{max}}=\max_{i}z_{{\text{HCA}}_{:,i,:,:}} \end{array}$$ (8)

Next, we calculate the gradient magnitude *G* using the Sobel operator, Conv_*x*_ and Conv_*y*_, as:


$$\begin{array}{c} G=\sqrt{{\left({\text{Conv}}_{x}(M_{\text{avg}})\right)}^{2}+{\left({\text{Conv}}_{y}(M_{\text{avg}})\right)}^{2}} \end{array}$$ (9)

We then concatenate the gradient *G* with the maximum feature map and apply a convolution to generate attention:


$$\begin{array}{c} A^{\prime }={\text{Conv2d}}_{7\times 7}\left(\text{Cat}[G;M_{\text{max}}]\right) \end{array}$$ (10)

The boundary-enhanced attention is further refined through boundary enhancement convolution:


$$\begin{array}{c} A^{\prime \prime }=\text{BoundaryConv}\left(A^{\prime }\right) \end{array}$$ (11)

Finally, the two attention maps are summed to obtain the final skin spatial attention map. The features are weighted and combined with residual connections to get the final output $z_{\text{SSA}}\in {\mathbb{R}}^{B\times 512\times 7\times 7}$:


$$\begin{array}{c} z_{\text{SSA}}={\text{Conv2d}}_{1\times 1}(x)+\left(z_{\text{HCA}}⊗\sigma \left(A^{\prime }+A^{\prime \prime }\right)\right) \end{array}$$ (12)

Skin lesion images often contain noise from hair, bubbles, etc. In few-shot learning scenarios, such noise is more likely to interfere with the model’s generalization performance. The SSA module guides the network using gradient information to distinguish real lesion boundaries from image noise, reducing erroneous attention to noise features and improving the robustness of few-shot learning.

### Architecture details

AMCANet employs the first six feature extraction layers of ConvNeXt-Tiny as the backbone, which extracts discriminative features from input skin lesion images with a resolution of 224 × 224. The backbone outputs a feature map with a spatial size of 14 × 14 and a channel dimension of 384, laying a solid foundation for subsequent attention-based feature optimization.

Adaptive Multi-Scale Convolution Module (AMSC) is designed to capture multi-scale contextual features via four parallel convolutional branches, with consistent input and output channel counts (384). The branch configurations are specified as follows: Branch 1 adopts 1 × 1 convolution (dilation = 1, padding = 0); Branch 2 utilizes 3 × 3 convolution (dilation = 1, padding = 1); Branch 3 applies 3 × 3 convolution (dilation = 2, padding = 2); Branch 4 employs 3 × 3 convolution (dilation = 4, padding = 4). For adaptive weight assignment, the output of each branch is fed into a weight generator after global average pooling (GAP). The generator consists of two fully connected (FC) layers with a compression ratio of 16 and a Softmax activation layer, generating four learnable scale weights. Multi-scale features are then weighted and fused to aggregate comprehensive contextual information, suppressing trivial features and highlighting critical lesion-related patterns.

Hierarchical Channel Attention Module (HCA) performs channel-wise feature recalibration by modeling hierarchical channel dependencies. It first conducts adaptive average pooling with kernel sizes 1 × 1, 2 × 2, and 4 × 4 on input features, yielding three resolution-differentiated feature descriptors. Each descriptor is flattened and forwarded through two FC layers: the first FC layer compresses the channel dimension to 1/16 of the original, and the second layer restores it to the initial channel count, generating three hierarchical channel weight maps. These hierarchical weights are element-wise summed and averaged, followed by a Sigmoid activation to generate the final channel attention map. The recalibrated features are obtained by multiplying the original features with the attention map, enhancing the representation of task-relevant channels.

Skin Spatial Attention Module (SSA) first computes the channel-wise average map *M*_avg_ and max map *M*_max_ to aggregate spatial information. A 3 × 3 Sobel operator (padding = 1) is applied to *M*_avg_ to extract horizontal and vertical gradient maps, denoted as *G*, highlighting lesion boundary features. The gradient map *G* and max map *M*_max_ are concatenated into a 2-channel feature tensor, which is processed by a 7 × 7 convolution (padding = 3) and Sigmoid activation to generate the initial spatial attention map. To refine boundary details, two consecutive 3 × 3 convolutions (padding = 1) with a ReLU activation in between are adopted for boundary enhancement, producing the refined attention map. The final spatial attention map is the sum of the initial and refined maps, which is multiplied with the original features and integrated via a residual connection to preserve shallow feature information.

The complete data flow of AMCANet is structured as follows: Input 224 × 224 skin lesion images are fed into the ConvNeXt-Tiny backbone to acquire 14 × 14 × 384 features. The features are sequentially processed by the AMSC module, a 1 × 1 convolution (input = 384, output = 512, stride = 2) for spatial downsampling to 7 × 7 and channel expansion, the HCA module, and the SSA module, outputting 7 × 7 × 512 refined features. Subsequently, the refined features are flattened via GAP and forwarded to a two-layer FC classifier for final classification. The first FC layer maps 512 dimensions to 512 dimensions with a ReLU activation, and the second FC layer projects features to the number of lesion categories. Dropout layers with ratios of 0.5 and 0.3 are inserted between the two FC layers respectively to mitigate overfitting. Notably, all convolutional layers (except specified ones) are followed by batch normalization (BN) and ReLU activation to stabilize training and accelerate convergence.

### Optimization

AMCANet adopts a hybrid architecture combining a backbone network and dedicated modules, balancing general feature extraction capabilities with skin lesion-specific optimization. The design of AMCANet follows the residual connection principle to ensure the stability of gradient propagation while allowing deep integration of dedicated modules. For skin lesion classification, we first use Global Average Pooling (GAP) to obtain the dimensionality reduction results of *z*_SSA_, and then pass it through a neural network classifier to obtain the predicted skin lesion results $\hat{Y}$, as shown below:


$$\begin{array}{c} \hat{Y}=\text{MLP}\left({\text{GAP}}_{1}(z_{\text{MSA}})\right)\in {\mathbb{R}}^{B\times N} \end{array}$$ (13)

where *N* represents the number of skin lesion categories. Finally, we use cross-entropy loss as the objective function, with the loss calculated as:


$$\begin{array}{c} ℒ=\frac{1}{B}\sum_{i=1}^{B}Y_{i}\cdot \log\left({\hat{Y}}_{i}\right) \end{array}$$ (14)

where *Y* is the truth label. After obtaining the loss, the model is optimized using gradient descent.

## Experiments

### Dataset

To validate the effectiveness of the proposed method, we conduct a comprehensive evaluation on the HAM10000 [17] and PAD-UFES-20 [22] datasets. The HAM10000 dataset contains approximately 10,015 dermoscopic images covering various common pigmented skin lesions. These images were collected from skin cancer clinics at the Medical University of Vienna in Austria and the University of Queensland in Australia over a span of 20 years. The HAM10000 dataset consists of 7 categories: Actinic Keratosis and Intraepithelial Carcinoma / Bowen’s Disease (AKIEC), Basal Cell Carcinoma (BCC), Benign Keratosis-like Lesions (BKL), Dermatofibroma (DF), Melanoma (MEL), Melanocytic Nevus (NV), and Vascular Lesions (VASC). The PAD-UFES-20 dataset contains 2,298 skin disease images from 1,373 patients, captured using various smartphones. This dataset includes 6 categories, 3 of which are skin cancers: Basal Cell Carcinoma (BCC), Squamous Cell Carcinoma (SCC), and Melanoma (MEL), while the other 3 are skin diseases: Actinic Keratosis (ACK), Seborrheic Keratosis (SEK), and Nevus (NEV). For the few-shot setting, we use a standard split of the number of samples per class, which the 10-shot setting means that each class only has 10 samples in the training set. The basic statistics of both datasets are shown in Table 1.

**Table 1:** Dataset statistics. Where Val num and Test num represents the numbers of the samples respectively, Max num and Min num denote the largest and smallest number of category samples, respectively, and balance(num) is the ratio between the maximum and minimum number of category samples.

Dataset	Few-Shot setting	Val num	Test num	Category num	Max num	Min num	Balance (num)
HAM10000	10/20/50-shot	10	9955	7	6305(NV)	103(DF)	∼61:1
PAD-UFES-20	10/20-shot	10	2238	6	845(BCC)	52(MEL)	∼16:1

### Baselines

We selected 8 advanced skin lesion classification models as baseline comparisons. ConvNeXt [32] is a pure convolutional neural network architecture that integrates design concepts from Vision Transformers and achieves optimal results. DeepCNN [25] employs a synergistic design that strengthens features through preprocessing and enables efficient learning with a lightweight model, achieving precise skin lesion classification. DeepIL [33] utilizes deep intrinsic learning methods to accurately classify multiple categories of skin lesions. BIIGMANet [23] adopts multi-head independent-guided channel attention and simultaneously uses the Hilbert-Schmidt Independence Criterion to enforce neuron independence, enhancing feature diversity. It also integrates a saliency-driven mechanism that selectively perturbs non-significant vectors to suppress background activations, preventing the model from relying on static background cues. DPS [26] applies deep pixel-level supervision with constant mappings and deep mask pixel-level supervision using segmentation masks to perform skin lesion classification. HybridConvNeXt [14] utilizes CNN methods to extract local patterns and fine texture features, while using the ViT structure to capture long-range relationships between skin cancer regions. MATNet [15] is composed of multi-scale triplet spatial attention and multi-scale triplet channel attention sequences, learning detailed feature relationships across space and channel dimensions at different scales, enriching feature representations. MuRANet [16] systematically integrates a series of attention mechanisms in a step-by-step manner, effectively enhancing feature extraction and contextual understanding of skin lesion images.

### Implementation details

In terms of experimental implementation, we implemented the proposed Adaptive Multi-Scale Convolutional Attention Network (AMCANet) using the PyTorch [34] deep learning framework. The model uses ConvNeXt-Tiny [32] as the backbone network, utilizing only its first six feature extraction layers as the base feature extractor, followed by the AMCANet we designed for deep feature optimization. The input images are resized to a uniform resolution of 224 × 224. During training, we employed the Adam [35] optimizer, with an initial learning rate of 1 × 10^−4^ and weight decay set to 1 × 10^−4^. The learning rate scheduler adopts a cosine annealing with warm restarts strategy, with an initial period of *T*_0_ = 10 and a multiplier factor of 2. The training batch size is 10, the testing batch size is 50, and the maximum number of training epochs is set to 500. An early stopping mechanism is used, where training is terminated early if the validation accuracy does not improve for 20 consecutive epochs. Throughout the experiment, the data in the training, validation, and test sets do not overlap. The loss function is the cross-entropy loss. All experiments are conducted on an NVIDIA RTX3090 GPU. To ensure reproducibility, we fixed the random seed, disabled the cuDNN benchmarking optimization feature, and enabled deterministic algorithms.

To ensure a fair comparison of all baseline models under identical conditions, we adopt the following rigorous experimental control protocols. All competing baseline methods are either re-implemented within our unified framework or employed from their officially released codes, and are trained and evaluated on exactly the same data splits. Specifically, we employ a fixed *K*-shot setting per class (*K* = 10, 20, 50), ensuring that the training, validation, and test sets are identical and mutually exclusive across all models. The backbone is not further pre-trained; instead, we use the provided pre-trained weights (https://download.pytorch.org/models/convnext_tiny-983f1562.pth). Except for the backbone, all models are trained from random initialization without any additional pre-trained weights, thereby avoiding unfair advantages introduced by pre-training. For the optimizer, we adopt the default optimizer of each baseline model according to its original paper or official repository, along with the corresponding learning rate and annealing strategy. In addition, hyperparameters including batch size and early stopping mechanism are kept consistent with those used in AMCANet. All experimental results are reported as the mean±standard deviation over five distinct random seeds to eliminate randomness from individual runs and improve statistical reliability.

For model evaluation, we adopt metrics including Accuracy (ACC), F1-score, Recall, and Precision, whose mathematical definitions are given as follows:


$$\begin{array}{c} \text{ACC}=\frac{TP+TN}{TP+TN+FP+FN} \end{array}$$ (15)

$$\begin{array}{c} \text{F1-score}=\frac{2\times (\text{Recall}\times \text{Precision})}{\text{Recall}+\text{Precision}} \end{array}$$ (16)

$$\begin{array}{c} \text{Recall}=\frac{TP}{TP+FN} \end{array}$$ (17)

$$\begin{array}{c} \text{Precision}=\frac{TP}{TP+FP} \end{array}$$ (18)

where *TP* represents the number of samples correctly predicted as positive class, *TN* the number of samples correctly predicted as negative class, *FP* the number of samples incorrectly predicted as positive class, and *FN* the number of samples incorrectly predicted as negative class.

### Numerical results and discussion

#### Numerical results.

The comparative experimental results are presented in Tables 2 and 3. The proposed AMCANet yields state-of-the-art performance on two few-shot skin lesion classification tasks, namely HAM10000 and PAD-UFES-20, and significantly outperforms existing baseline models across a multitude of evaluation metrics.

**Table 2:** Results (%) of few-shot skin lesion classification for HAM10000. The best performance is in bold, the second best performance is underlined, and ▴ is the improvement over the best baseline.

a	ACC			F1-score		
Methods	10-shot	20-shot	50-shot	10-shot	20-shot	50-shot
ConvNeXt	14.70 ± 5.50	29.26 ± 10.14	41.30 ± 6.85	10.16 ± 2.16	17.90 ± 3.41	23.97 ± 2.11
DeepCNN	36.60 ± 3.38	47.56 ± 2.71	43.35 ± 3.84	19.69 ± 1.06	24.40 ± 1.46	24.42 ± 1.91
DeepIL	35.10 ± 9.37	51.44 ± 6.86	50.65 ± 2.71	16.53 ± 4.34	25.61 ± 3.49	30.06 ± 0.87
BIIGMANet	5.80 ± 3.96	12.46 ± 13.15	31.62 ± 22.63	2.76 ± 2.07	4.8 ± 3.45	9.26 ± 6.42
DPS	35.17 ± 11.04	30.36 ± 2.80	48.99 ± 5.49	16.51 ± 2.54	18.36 ± 2.18	24.96 ± 1.85
HybridConvNeXt	34.00 ± 0.49	33.33 ± 6.38	34.14 ± 3.14	17.37 ± 1.08	18.94 ± 1.49	20.87 ± 1.39
MATNet	43.25 ± 5.96	44.31 ± 4.66	52.72 ± 2.04	18.24 ± 3.56	23.03 ± 2.33	30.13 ± 1.08
MuRANet	44.25 ± 4.32	45.41 ± 2.04	44.76 ± 5.32	15.06 ± 2.10	17.21 ± 1.92	15.53 ± 2.02
**AMCANet**	**47.28 ± 3.38**	**57.83 ± 2.35**	**61.19 ± 3.44**	**26.60 ± 1.91**	**34.35 ± 0.81**	**41.76 ± 4.45**
▴	3.03	6.39	8.47	6.91	8.74	11.63
	**Recall**			**Precision**		
**Methods**	**10-shot**	**20-shot**	**50-shot**	**10-shot**	**20-shot**	**50-shot**
ConvNeXt	26.61 ± 2.80	32.15 ± 2.24	38.92 ± 0.77	22.86 ± 2.19	24.91 ± 2.36	28.68 ± 0.25
DeepCNN	30.69 ± 0.66	35.75 ± 0.82	39.67 ± 1.6	23.92 ± 0.55	25.52 ± 0.79	26.72 ± 1.45
DeepIL	30.31 ± 3.95	40.96 ± 2.65	44.45 ± 0.63	23.78 ± 3.92	27.48 ± 1.19	29.79 ± 0.67
BIIGMANet	15.85 ± 2.06	16.62 ± 4.32	18.05 ± 2.60	2.98 ± 3.00	6.53 ± 5.17	10.24 ± 6.28
DPS	25.70 ± 3.56	33.97 ± 4.32	41.44 ± 4.56	20.62 ± 1.09	22.36 ± 1.17	26.65 ± 1.76
HybridConvNeXt	29.65 ± 1.70	32.02 ± 1.06	37.45 ± 1.56	21.04 ± 0.48	21.37 ± 0.55	23.15 ± 0.31
MATNet	27.38 ± 4.47	38.71 ± 1.74	47.07 ± 1.41	21.29 ± 2.49	24.61 ± 1.60	30.39 ± 0.54
MuRANet	22.68 ± 1.09	24.76 ± 1.65	23.18 ± 1.08	19.02 ± 2.94	20.13 ± 1.67	18.78 ± 1.65
**AMCANet**	**42.16 ± 1.22**	**53.61 ± 1.49**	**63.90 ± 2.87**	**27.39 ± 1.41**	**34.89 ± 1.05**	**40.54 ± 1.59**
▴	11.47	12.65	16.83	3.47	7.41	10.15

**Table 3:** Results (%) of few-shot skin lesion classification for PAD-UFES-20.

	ACC		F1-score		Recall		Precision	
Methods	10-shot	20-shot	10-shot	20-shot	10-shot	20-shot	10-shot	20-shot
ConvNeXt	24.01 ± 2.09	28.97 ± 1.91	19.44 ± 2.62	24.00 ± 2.41	29.61 ± 2.26	36.09 ± 0.95	27.29 ± 1.14	32.82 ± 2.68
DeepCNN	25.32 ± 1.10	28.35 ± 4.60	18.31 ± 1.36	20.00 ± 1.35	26.33 ± 2.59	26.06 ± 1.74	20.21 ± 1.14	21.94 ± 1.38
DeepIL	30.51 ± 5.34	31.60 ± 3.69	19.28 ± 1.24	22.63 ± 2.86	29.36 ± 3.12	34.27 ± 1.89	23.03 ± 2.21	27.98 ± 3.44
BIIGMANet	20.35 ± 8.44	19.69 ± 8.89	8.89 ± 1.22	8.90 ± 3.73	18.55 ± 3.40	18.71 ± 3.92	11.89 ± 6.91	8.54 ± 1.90
DPS	22.69 ± 4.53	25.78 ± 2.88	18.80 ± 2.05	22.88 ± 1.78	27.33 ± 1.52	33.81 ± 1.80	23.55 ± 0.97	26.38 ± 1.47
HybridConvNeXt	20.26 ± 1.53	20.96 ± 2.35	17.35 ± 0.83	17.72 ± 1.95	25.35 ± 0.56	25.66 ± 1.50	21.77 ± 0.80	21.34 ± 0.73
MATNet	24.53 ± 5.63	22.57 ± 7.38	16.62 ± 1.29	17.66 ± 7.66	22.74 ± 3.03	27.66 ± 8.35	18.35 ± 2.12	22.74 ± 7.93
MuRANet	18.92 ± 7.14	27.21 ± 3.66	10.43 ± 3.68	15.42 ± 1.33	18.01 ± 1.59	18.49 ± 0.98	11.32 ± 3.05	15.79 ± 1.24
**AMCANet**	**38.38 ± 2.27**	**44.36 ± 3.40**	**30.52 ± 1.11**	**37.54 ± 1.24**	**37.22 ± 2.55**	**46.89 ± 1.03**	**32.30 ± 1.48**	**38.49 ± 1.01**
▴	7.87	12.76	11.08	13.54	7.61	10.80	5.01	5.67

On the HAM10000 dataset (Table 2), AMCANet achieves classification accuracies of 47.28%, 57.83%, and 61.19% under the 10-shot, 20-shot, and 50-shot settings, respectively. Relative to the top-performing baseline models (MuRANet, DeepIL, MATNet), it delivers accuracy gains of 3.03%, 6.39%, and 8.47% correspondingly. For the more challenging F1-score metric, the superiority of AMCANet becomes even more prominent: it records F1-scores of 26.60%, 34.35%, and 41.76% under the three few-shot settings, representing substantial improvements of 6.91%, 8.74%, and 11.63% over the best-performing baselines. Notably, in the 50-shot scenario, the recall rate of AMCANet reaches 63.90%, surpassing the optimal baseline (DPS) by a remarkable 16.83 percentage points, which fully validates the exceptional performance of the proposed model in few-shot skin lesion classification tasks.

On the PAD-UFES-20 dataset (Table 3), AMCANet further demonstrates robust generalization capability. Under the 10-shot and 20-shot configurations, it attains accuracies of 38.38% and 44.36%, with respective increments of 7.87 and 12.76 percentage points compared to the strongest baseline models. The enhancements in F1-score are even more pronounced, registering values of 30.52% and 37.54%, which exceed those of the top baselines (DeepIL, ConvNeXt) by 11.08 and 13.54 percentage points, respectively. These results further corroborate the robustness and generalization capacity of AMCANet under diverse data acquisition conditions, including smartphone-captured images in the PAD-UFES-20 dataset, thereby verifying its promising application potential in real-world clinical settings.

## Discussion

The superior performance of AMCANet is predominantly attributed to the synergistic integration of three bespoke core modules tailored to the inherent characteristics of skin lesion imagery. Primarily, the Adaptive Multi-Scale Convolution (AMSC) module mitigates the pronounced heterogeneity in skin lesion sizes via dynamic receptive field modulation. When processing diverse lesions spanning minute macules to extensive plaques within the HAM10000 dataset, this module eliminates the incorporation of extraneous contextual information, enabling precise capture of both global architectural features and local fine-grained details of lesions across varying scales, thereby laying a robust foundation for subsequent fine-grained lesion classification. Secondarily, the Hierarchical Channel Attention (HCA) module facilitates effective fusion of multi-resolution semantic cues by evaluating channel-wise significance across distinct hierarchical feature maps. This not only augments the model’s capacity to extract discriminative pathological features critical for lesion identification, but also bolsters feature robustness against inter-image style variations induced by disparate imaging devices and acquisition protocols in the PAD-UFES-20 dataset, ensuring the model prioritizes core lesion-specific semantic information over irrelevant imaging artifacts. Ultimately, the Skin Spatial Attention (SSA) module serves as a pivotal component of the framework. By leveraging image gradient information to accentuate lesion boundaries and fine local textural features, the SSA module is indispensable for differentiating clinically ambiguous and morphologically similar lesion subtypes. Notably, in data-scarce scenarios with extremely limited annotated samples, the SSA module steers the network to concentrate on clinically salient boundary and textural biomarkers rather than background noise, markedly enhancing the model’s few-shot learning capability and cross-domain generalization performance. Concretely, the remarkable 13.54% improvement in F1-score achieved by the proposed AMCANet under the 20-shot few-shot learning setting on the PAD-UFES-20 dataset substantiates the efficacy of this module.

In comparison with baseline architectures, conventional convolutional models including ConvNeXt and DeepCNN rely on fixed-size convolutional kernels and natural image-oriented designs, which fail to adequately accommodate the extreme morphological diversity and localized pathological specificity of skin lesions. While attention-augmented networks such as BIIGMANet and MATNet enhance feature expressiveness to a certain degree, their generic attention mechanisms exhibit insufficient sensitivity to the pivotal skin lesion attributes, particularly lesion boundaries and subtle textural patterns that carry critical diagnostic value. In contrast, AMCANet unifies adaptive scale perception (AMSC), hierarchical channel refinement (HCA), and skin-specific spatial feature enhancement (SSA) to realize high-precision pathological feature representation. This tailored design enables the model to sustain superior classification accuracy and robust generalization even in few-shot scenarios, validating its distinct superiority for dedicated skin lesion analysis tasks.

### Confusion analysis

**Fig 2:**
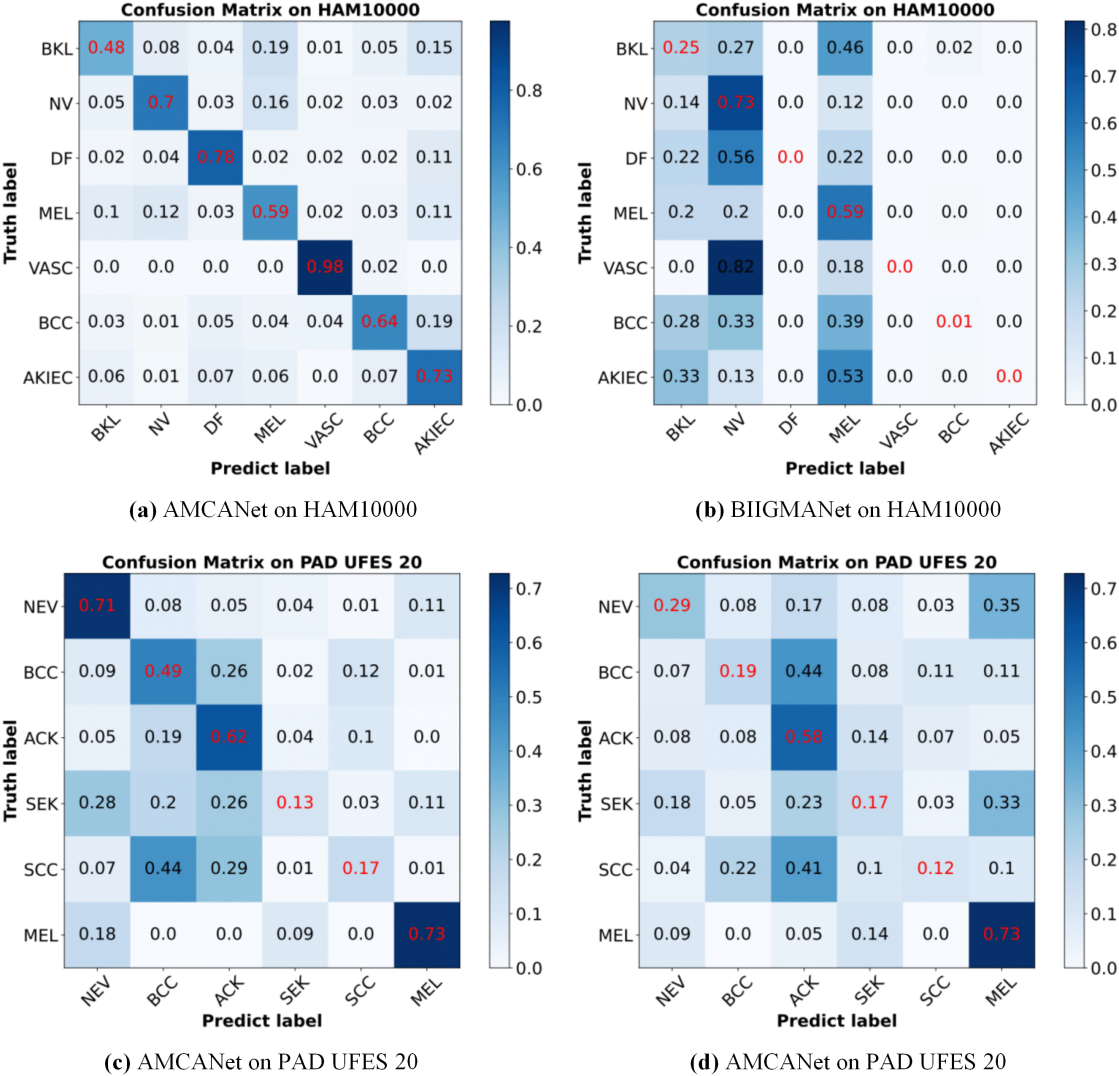
Confusion matrix results. The diagonal represents the probability of correct predictions, while the rest represent the probabilities of predicting a certain category as another category.

To intuitively understand the model’s classification performance on different skin lesion categories, we present a classification confusion matrix analysis. The experiments in this section were conducted on the HAM10000 and PAD-UFES-20 datasets under the 50-shot and 20-shot settings, respectively, with the results shown in Fig 2. On the HAM10000 dataset, the confusion matrix of AMCANet (see Fig 2a) shows that it maintains high diagonal probabilities for most categories, indicating good intra-class consistency and classification confidence. For example, for two lesion types with relatively unique morphologies, vascular lesions (VASC) and dermatofibroma (DF), AMCANet achieved classification accuracies of 98% and 78%, respectively, significantly outperforming the strongest baseline model BIIGMANet (see Fig 2b, where VASC was severely misclassified as NV with a probability of 82%). This result suggests that AMCANet, by enhancing boundaries and local structures through its skin spatial attention module, effectively captures discriminative features of lesions, reducing misclassification caused by visual similarity. In more challenging common lesion categories, AMCANet also demonstrates stronger discriminative power. For example, for the clinically confusing melanoma (MEL) and melanocytic nevus (NV), AMCANet achieved an accuracy of 59% for MEL, with much lower confusion compared to BIIGMANet (where MEL was misclassified as NV and BKL with probabilities of 20% each). Similarly, for actinic keratosis/in situ carcinoma (AKIEC) and basal cell carcinoma (BCC), AMCANet achieved correct classification probabilities of 73% and 64%, while BIIGMANet struggled to differentiate these two lesions effectively (with 0 accuracy for AKIEC and only 1% for BCC). This improvement is primarily attributed to the dynamic adaptation of lesion size and morphology in the adaptive multi-scale convolution module, as well as the hierarchical channel attention module’s ability to select discriminative features across multiple levels, enabling the model to better distinguish lesions with similar textures but different pathological significance.

On the PAD-UFES-20 dataset, the comparison of AMCANet (see Fig 2c) and DeepIL (see Fig 2d) confusion matrices further validates its generalization performance. AMCANet achieved classification accuracies of 71% and 73% for nevus (NEV) and melanoma (MEL), respectively, while DeepIL achieved only 29% and 73%, showing a clear advantage for AMCANet in NEV classification. Notably, for the clinically confused basal cell carcinoma (BCC), squamous cell carcinoma (SCC), and actinic keratosis (ACK), AMCANet still maintained relatively robust classification performance, with accuracies of 49%, 17%, and 62%, significantly outperforming DeepIL (with corresponding accuracies of 19%, 12%, and 58%). This suggests that AMCANet maintains high classification reliability even on cross-device and heterogeneously collected image data. Comprehensive analysis of the classification confusion matrices across the two datasets shows that AMCANet not only leads in overall metrics but also exhibits stronger robustness in inter-class differentiation. The synergistic effects of its core modules reduce inter-class confusion and enhance the model’s sensitivity to subtle differences in skin lesions, thereby achieving more accurate and reliable lesion classification under few-shot conditions. This provides an important technical foundation for its application in clinical-assisted diagnosis.

### Ablation studies

#### Module-level ablation analysis.

We first validate the individual contributions of the three core modules: AMSC, HCA, and SSA. Table 4 presents the performance variations by removing one single module, two combined modules, and all modules sequentially based on the complete model. The full AMCANet achieves the highest ACC and F1-score across all experimental settings, verifying the rationality of the collaborative design of the three modules. Under the 20-shot setting on the PAD-UFES-20 dataset, removing SSA (w/o SSA) reduces ACC from 44.36% to 43.49% and F1-score from 37.54% to 36.58%. Despite the slight numerical drop, the SSA module plays a vital role in stabilizing feature extraction by enhancing lesion boundaries and local textures via gradient information, considering the challenges of PAD-UFES-20 images (captured by smartphones with complex backgrounds and blurred lesion edges). Under the harsher 10-shot setting, eliminating SSA causes ACC to drop from 38.38% to 35.92% and F1-score to plummet from 30.52% to 20.60%, highlighting the significance of boundary enhancement in mitigating overfitting and noise interference under extremely few-shot conditions. Similarly, removing SSA leads to a minor ACC decline and a slight F1 drop from 26.60 to 26.20 in the 10-shot setting on HAM10000. Overall, the SSA module delivers more prominent performance gains in low-data scenarios and complex background environments.

**Table 4:** Results (%) of ablation studies. Where AMSC, HCA and SSA represent adaptive multi-scale convolution module, hierarchical channel attention and skin-spatial attention respectively. w/o means to remove a certain item, and ALL means to remove all items.

Module	HAM10000						PAD-UFES-20			
	10-shot		20-shot		50-shot		10-shot		20-shot	
	ACC	F1-score	ACC	F1-score	ACC	F1-score	ACC	F1-score	ACC	F1-score
AMCANet	**47.28 ± 3.38**	**26.60 ± 1.91**	**57.83 ± 2.35**	**34.35 ± 0.81**	61.19 ± 3.44	**41.76 ± 4.45**	**38.38 ± 2.27**	**30.52 ± 1.11**	**44.36 ± 3.40**	**37.54 ± 1.24**
w/o AMSC	41.96 ± 2.44	25.66 ± 3.65	46.41 ± 7.19	29.35 ± 7.63	58.09 ± 3.18	41.03 ± 4.11	29.34 ± 6.00	26.37 ± 3.77	35.41 ± 5.86	31.57 ± 5.19
w/o HCA	46.92 ± 4.16	25.89 ± 1.84	56.52 ± 4.03	34.11 ± 3.54	**61.88 ± 5.80**	41.22 ± 4.41	37.20 ± 2.71	29.05 ± 0.98	44.33 ± 3.56	37.04 ± 2.70
w/o SSA	47.01 ± 3.06	26.20 ± 1.67	49.60 ± 6.33	33.82 ± 2.71	60.21 ± 2.77	40.16 ± 1.69	35.92 ± 5.17	20.60 ± 2.71	43.49 ± 3.04	36.58 ± 2.81
w/o AMSC+HCA	43.46 ± 6.96	26.28 ± 3.21	56.27 ± 4.68	33.54 ± 0.54	56.88 ± 5.49	36.30 ± 5.20	34.83 ± 1.88	28.59 ± 2.58	42.34 ± 6.87	37.26 ± 4.22
w/o AMSC+SSA	41.52 ± 8.49	23.67 ± 3.00	46.05 ± 10.03	29.25 ± 9.88	53.49 ± 6.00	40.42 ± 3.87	31.05 ± 6.71	26.29 ± 4.30	39.55 ± 5.26	35.24 ± 4.74
w/o HCA + SSA	42.63 ± 4.65	25.52 ± 2.26	57.23 ± 5.11	33.22 ± 2.57	60.22 ± 2.15	40.88 ± 2.05	35.72 ± 6.60	30.09 ± 3.41	42.77 ± 4.76	37.24 ± 2.43
w/o All	40.61 ± 8.92	23.55 ± 2.69	45.25 ± 3.99	29.17 ± 1.85	53.90 ± 5.11	34.46 ± 5.07	30.51 ± 3.75	25.65 ± 3.28	35.43 ± 3.23	30.57 ± 3.23

Removing either AMSC or HCA alone incurs performance degradation. For instance, in the 20-shot setting on HAM10000, eliminating AMSC reduces ACC from 57.83% to 46.41% and F1-score from 34.35% to 29.35%; removing HCA lowers ACC to 56.52% and F1-score to 34.11%. When both AMSC and HCA are removed (w/o AMSC+HCA), the performance deteriorates further, with ACC dropping to 56.27% and F1-score to 33.54%. Under the 50-shot setting, the negative impact of dual-module removal is more pronounced: ACC falls from 61.19% to 56.88% and F1-score from 41.76% to 36.30%. This confirms that the multi-scale adaptive receptive field provided by AMSC and the hierarchical channel recalibration enabled by HCA are mutually complementary, jointly modeling the complex morphological features and multi-level semantic information of skin lesions. After removing all three modules (w/o All), the model achieves the lowest performance across all metrics. For example, in the 50-shot setting on HAM10000, ACC is merely 53.90% and F1-score only 34.46%, far below the complete model, which validates the necessity of integrating the three modules.

#### Intra-module component ablation analysis.

To further explore the optimality of the internal design of each module, we conduct component replacement experiments on AMSC, HCA, and SSA respectively, with the results shown in Table 5.

**Table 5:** Module internal component ablation results (%). The variant column represents only used modules, where BE represents boundary enhancement. In the AMSC module, three variants represent the replacement of multi-scale adaptive fusion. In the HCA module, six variants represent substitutions to hierarchical pooling. In SSA module, three variants represent the replacement of the way attention is calculated.

Module	Variant	HAM10000						PAD-UFES-20			
		10-shot		20-shot		50-shot		10-shot		20-shot	
		ACC	F1-score	ACC	F1-score	ACC	F1-score	ACC	F1-score	ACC	F1-score
AMSC	Default(AMCANet)	**47.28 ± 3.38**	**26.60 ± 1.91**	57.83 ± 2.35	**34.35 ± 0.81**	**61.19 ± 3.44**	41.76 ± 4.45	**38.38 ± 2.27**	**30.52 ± 1.11**	**44.36 ± 3.40**	37.54 ± 1.24
	Convolution 3×3	46.94 ± 4.52	26.04 ± 2.58	51.71 ± 1.79	33.96 ± 1.54	60.61 ± 2.34	**44.97 ± 3.64**	35.20 ± 4.52	30.12 ± 3.14	44.01 ± 6.73	**37.86 ± 4.78**
	Sum	44.55 ± 7.56	26.09 ± 2.19	**57.99 ± 3.82**	34.28 ± 1.95	60.52 ± 3.39	41.00 ± 3.02	34.97 ± 5.21	30.19 ± 5.69	42.23 ± 1.83	37.63 ± 1.71
	Mean	45.00 ± 7.71	26.55 ± 3.57	57.33 ± 8.04	33.72 ± 3.38	60.78 ± 4.28	41.97 ± 6.02	34.58 ± 2.73	30.51 ± 1.87	40.07 ± 3.50	37.36 ± 1.94
HCA	Default(AMCANet)	**47.28 ± 3.38**	**26.60 ± 1.91**	**57.83 ± 2.35**	**34.35 ± 0.81**	**61.19 ± 3.44**	**41.76 ± 4.45**	**38.38 ± 2.27**	30.52 ± 1.11	**44.36 ± 3.40**	37.54 ± 1.24
	Only 1×1 Pooling	44.44 ± 4.72	25.69 ± 2.34	48.19 ± 9.05	33.82 ± 4.79	59.69 ± 4.61	40.72 ± 3.40	34.17 ± 3.37	29.68 ± 2.19	40.01 ± 5.98	36.26 ± 2.51
	Only 2×2 Pooling	46.24 ± 3.27	26.45 ± 2.15	56.59 ± 7.77	33.53 ± 2.82	61.17 ± 4.96	41.27 ± 3.89	35.00 ± 6.76	29.55 ± 4.18	42.59 ± 3.89	37.51 ± 3.06
	Only 4×4 Pooling	46.08 ± 11.88	25.53 ± 4.26	51.89 ± 7.38	32.47 ± 5.32	60.78 ± 2.62	41.60 ± 1.47	37.54 ± 4.73	29.79 ± 1.90	41.74 ± 2.59	36.90 ± 2.21
	1×1 + 2×2 Pooling	46.41 ± 10.92	26.08 ± 2.66	56.72 ± 5.31	32.90 ± 3.05	60.08 ± 2.96	40.78 ± 2.42	37.68 ± 4.13	29.50 ± 3.84	41.16 ± 1.91	36.51 ± 1.36
	1×1 + 4×4 Pooling	42.65 ± 5.01	24.72 ± 2.53	54.48 ± 3.78	33.13 ± 2.81	60.27 ± 2.35	40.56 ± 3.57	37.80 ± 3.54	30.31 ± 1.09	43.96 ± 4.98	37.23 ± 3.44
	2×2 + 4×4 Pooling	46.40 ± 5.06	25.22 ± 2.07	55.51 ± 3.19	34.17 ± 1.71	60.64 ± 4.61	40.92 ± 2.86	38.20 ± 3.65	**31.38 ± 2.56**	44.11 ± 4.16	**37.62 ± 2.21**
SSA	Default(AMCANet)	**47.28 ± 3.38**	26.60 ± 1.91	**57.83 ± 2.35**	**34.35 ± 0.81**	**61.19 ± 3.44**	**41.76 ± 4.45**	**38.38 ± 2.27**	30.52 ± 1.11	**44.36 ± 3.40**	37.54 ± 1.24
	Only Max	46.88 ± 12.54	26.04 ± 4.89	53.80 ± 4.38	33.91 ± 2.19	60.04 ± 1.66	40.14 ± 1.64	36.52 ± 2.75	30.35 ± 2.00	43.60 ± 3.75	**37.73 ± 2.43**
	Only Avg	44.65 ± 7.80	25.91 ± 2.96	53.20 ± 4.61	34.16 ± 4.26	60.50 ± 3.78	40.91 ± 4.41	33.79 ± 2.49	**31.44 ± 0.91**	41.03 ± 4.31	36.36 ± 2.97
	w/o BE	47.00 ± 7.36	**26.80 ± 3.97**	56.65 ± 7.30	34.08 ± 2.49	57.36 ± 4.90	41.63 ± 3.29	35.53 ± 4.06	30.05 ± 1.94	44.32 ± 4.73	37.08 ± 2.85

For AMSC module, we compare the default adaptive weighted fusion strategy with three alternative schemes (3×3 convolution only, direct summation, average pooling). The default AMCANet attains the optimal performance under most settings, especially in the 10-shot setting on HAM10000 and 10-shot/20-shot settings on PAD-UFES-20, leading in both ACC and F1-score. For example, in the 10-shot setting on PAD-UFES-20, the default ACC of 38.38% surpasses 35.20%, 34.97%, and 34.58% of the other variants. Although the “summation” variant achieves a marginally higher ACC (57.99% vs. 57.83%) in the 20-shot setting on HAM10000, its F1-score is slightly lower; the “3×3 convolution only” variant yields an F1-score of 44.97% in the 50-shot setting on HAM10000, but its ACC of 60.61% is inferior to the default 61.19%. In summary, adaptive weighted fusion dynamically adjusts the contribution of multi-scale features based on sample characteristics, avoiding noise introduced by fixed fusion strategies.

For HCA module, We design six variants with different multi-scale pooling combinations (1±1 only, 2±2 only, 4±4 only, 1±1 + 2±2, 1±1 + 4±4, 2±2 + 4±4). The default HCA (integrating three pooling scales) achieves the best performance under most settings. For instance, in the 10-shot setting on HAM10000, the default ACC (47.28%) and F1-score (26.60%) outperform all variants; the default ACC of 38.38% also ranks first in the 10-shot setting on PAD-UFES-20. Notably, the “Only Avg” variant achieves an F1-score of 31.44% in the 10-shot setting on PAD-UFES-20, slightly higher than the default 30.52%, but its ACC is lower. Overall, multi-scale joint pooling captures global, regional and local information simultaneously, endowing channel attention with stronger hierarchy and richness, while single-scale pooling tends to lose partial semantic information and cause performance fluctuations.

For SSA module, We compare the default design (integrating max pooling, average pooling, Sobel gradient operator and boundary enhancement convolution) with three variants (max pooling only, average pooling only, removing boundary enhancement BE). The default SSA achieves optimal performance on nearly all metrics, particularly in the 10-shot settings on HAM10000 and PAD-UFES-20, with higher ACC and F1-score than the variants. For example, in the 10-shot setting on HAM10000, the default F1-score of 26.60% is superior to 26.04% (max pooling only) and 25.91% (average pooling only); in the 10-shot setting on PAD-UFES-20, the default ACC of 38.38% is significantly higher than 33.79% (average pooling only). Removing boundary enhancement (w/o BE) leads to universal performance degradation, e.g., ACC drops from 61.19% to 57.36% in the 50-shot setting on HAM10000, proving that gradient-guided boundary information is critical for spatial attention focusing on lesion regions.

In conclusion, both module-level and intra-module ablation studies consistently demonstrate that the designs of AMSC, HCA and SSA are elaborately optimized. Their default configurations present the optimal synergistic effect in addressing the challenges of skin lesion classification, including scale diversity, blurred boundaries, intra-class variation and few-shot learning constraints. These experimental results not only validate the indispensability of each module, but also confirm the rationality of internal component selection for the proposed framework.

### Qualitative analysis

**Fig 3:**
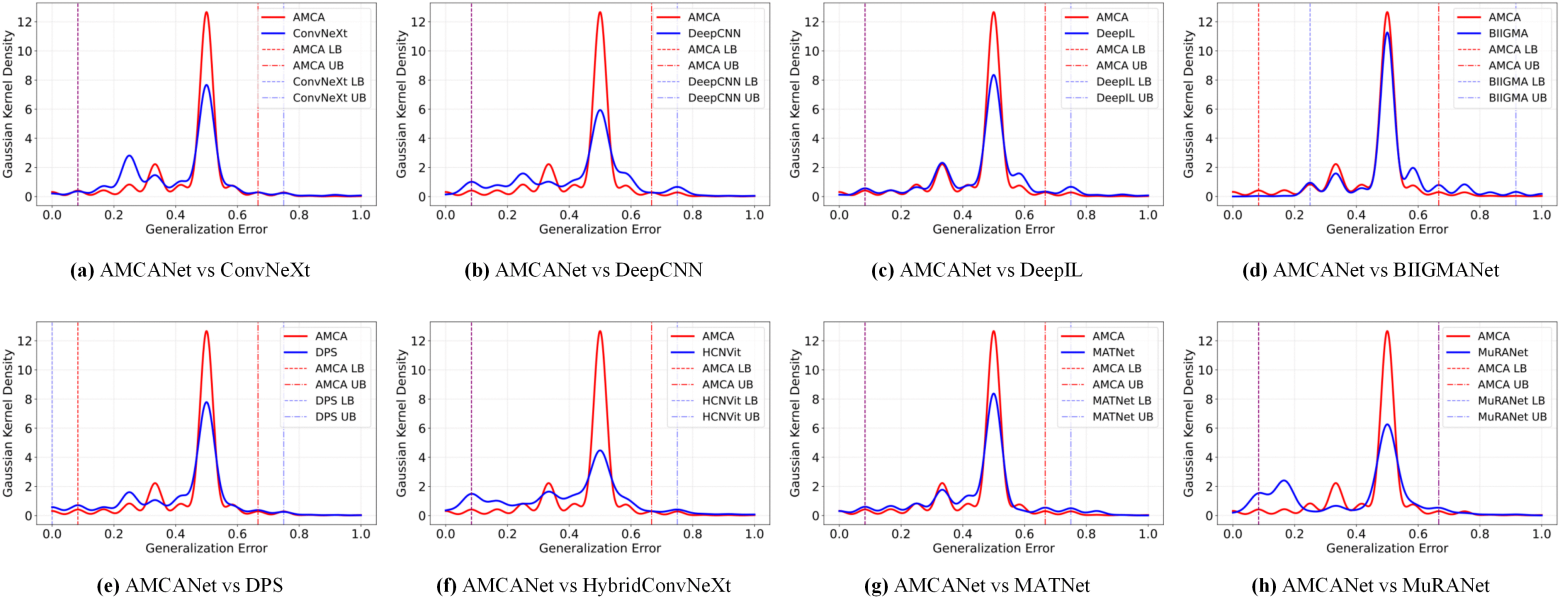
Upper and lower bounds on the generalization error. Where LB and UP denote the lower and upper bounds, respectively.

To observe and understand the underlying reasons for the effectiveness of AMCANet, we performed a qualitative analysis in this section, covering both generalization performance and feature representation. The qualitative experiments in this section were conducted on the HAM10000 dataset under the 50-shot setting. Fig 3 presents the qualitative results of the generalization error analysis (obtained using Gaussian Kernel Density [36] estimation). From this, it can be observed that the error curve of AMCANet (red) generally exhibits a sharper distribution shape in most comparisons, with the confidence interval (LB-UB) of its error distribution being narrower overall compared to baseline models (blue). This suggests that AMCANet demonstrates more stable predictive performance and lower generalization variance on the dataset. For example, in the generalization results of mainstream models such as DeepIL (Fig 3c), BIIGMANet (Fig 3d), and MATNet (Fig 3g), the upper bound (UB) of the generalization error is significantly higher than that of AMCANet, indicating that these models produce larger errors on unseen test samples. This phenomenon shows that AMCANet has stronger adaptability and reliability when dealing with unseen samples.

**Fig 4:**
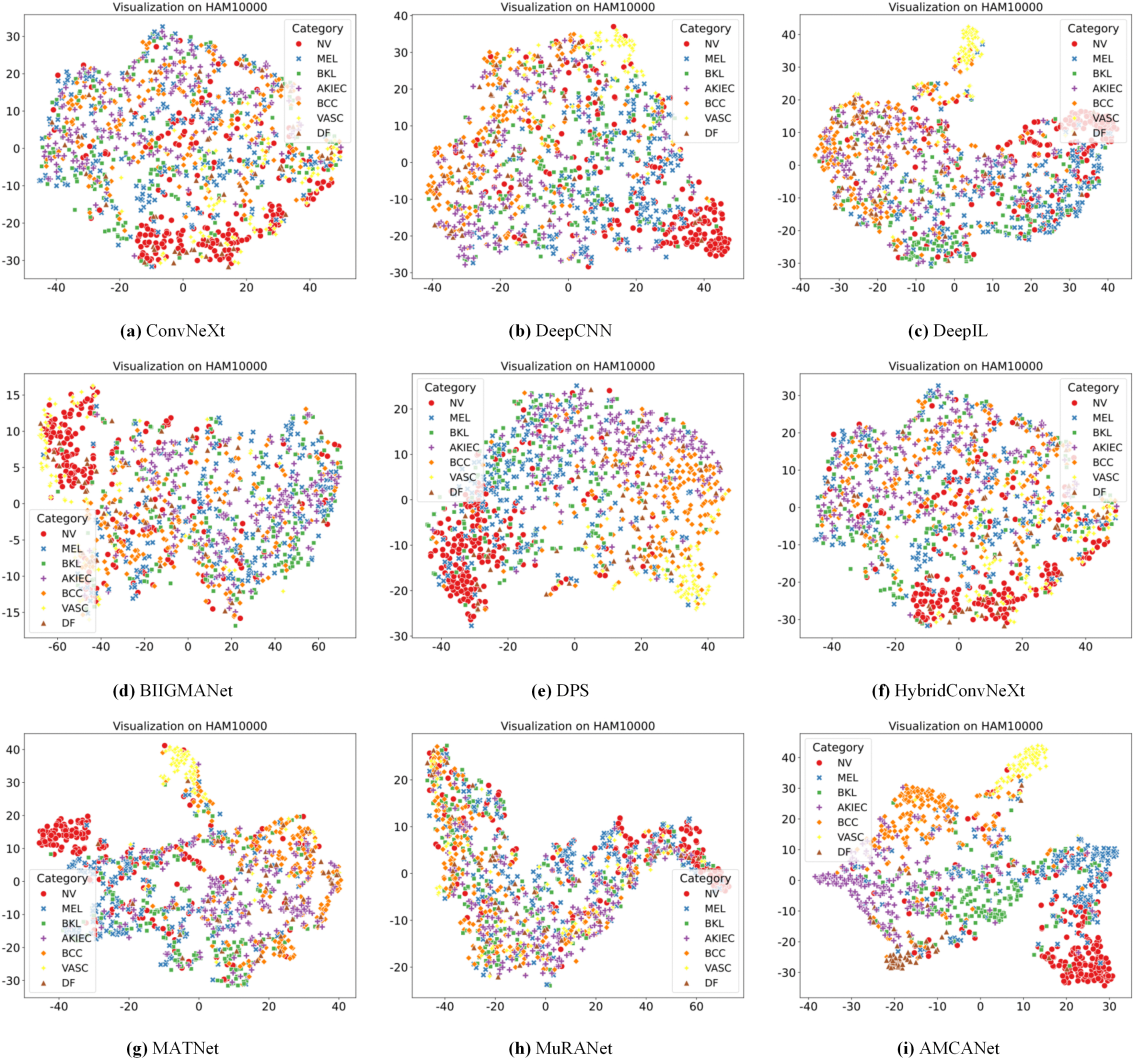
Visualization of the feature space representation. Different colors represent different classes, and the closer the features are to each other, the better the result is.

Fig 4 illustrates the feature space representation visualization of different models (t-SNE [37] dimensionality reduction plot), from which it can be seen that the features learned by AMCANet exhibit clearer inter-class separation and more compact intra-class clustering in the low-dimensional space. As shown in Fig 4, the feature projection of AMCANet (Fig 4i) shows that sample points of different categories (e.g., NV, BCC, VASC, DF, etc.) form relatively independent cluster structures with minimal inter-class overlap. In contrast, in the feature visualizations of baseline models such as DeepCNN (Fig 4b), DeepIL (Fig 4c), and BIIGMANet (Fig 4d), the sample points of different categories are more intermixed, especially between malignant lesions (e.g., MEL) and benign lesions (e.g., NV, BKL), where significant overlap exists. These results indicate that the features produced by AMCANet are more discriminative, resulting in more consistent feature representations for the same type of skin lesions, while being more distinct between different types of lesions.

By combining the qualitative analysis of generalization error and feature representation visualization, it can be concluded that AMCANet not only demonstrates more stable error control during the learning process but also achieves better inter-class separability and intra-class compactness in feature representations. This proves that AMCANet, in addressing the few-shot skin lesion classification task, not only leads in numerical metrics but also has significant advantages in learning robustness and feature independence.

### Attention visualization

**Fig 5:**
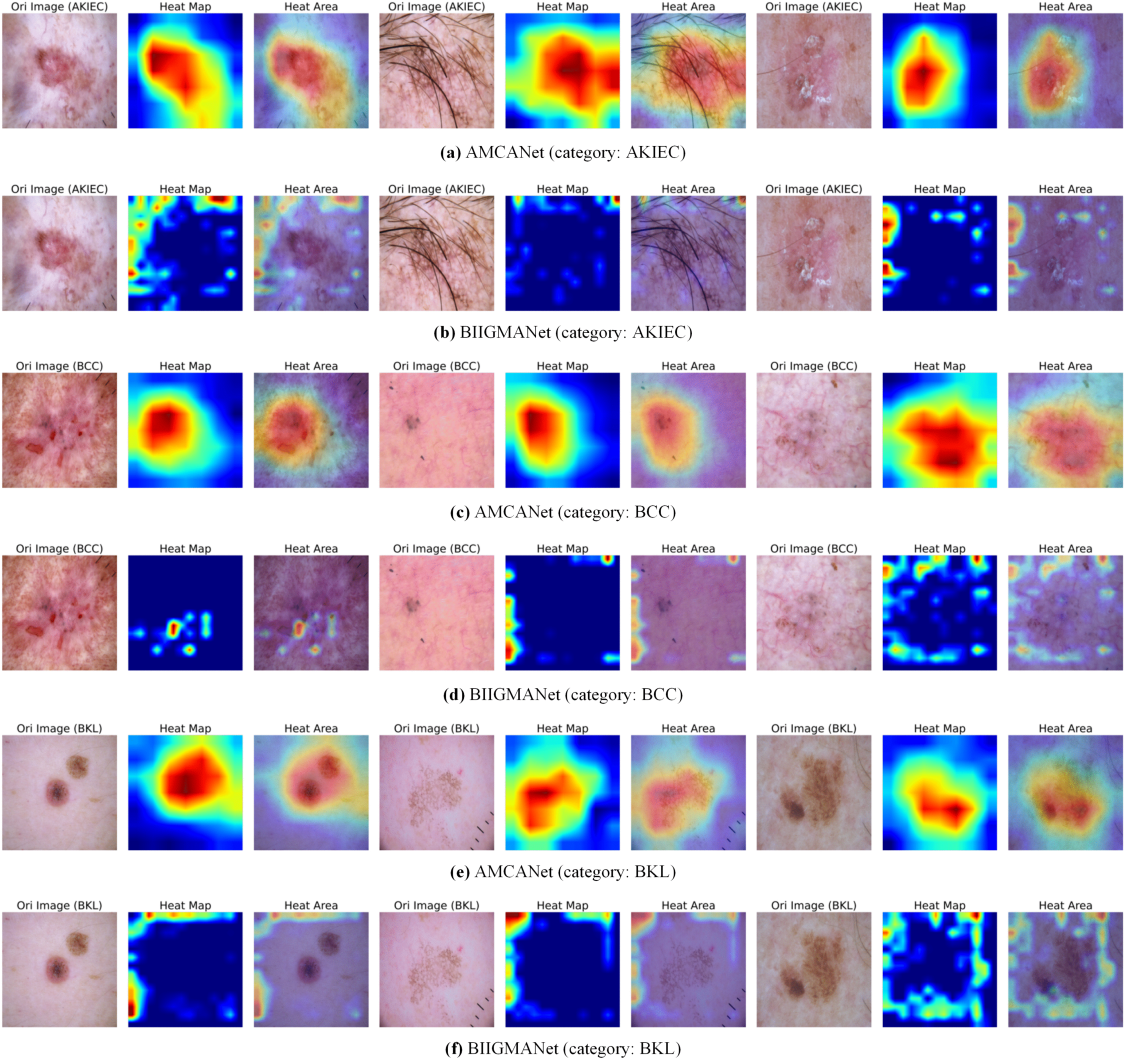
Heatmap visualizations using Grad-CAM [38] on HAM10000. The closer the color is to red, the more attention the model is paying to this region. **(a)**, **(c)**, and **(e)** are the results of AMCANet, while **(b)**, **(d)**, and **(f)** are the results of the baseline BIIGMANet.

To gain a deeper understanding of the interpretability and explainability of AMCANet, we plotted heatmaps of the images in the HAM10000 dataset. The purpose of this experiment was to validate whether the proposed AMCANet could effectively focus on the relevant regions of skin lesions. To highlight the differences, we compared it with the most robust baseline, BIIGMANet, under the 50-shot setting of the HAM10000 dataset, with the results shown in Fig 5. From the results, it is evident that AMCANet can effectively concentrate on the lesion areas and localize them, while BIIGMANet focuses on some unnecessary regions. This result significantly strengthens the credibility of skin lesion classification interpretations, where the ability to focus on the correct lesion regions is a necessary condition for accurate classification. Clearly, AMCANet demonstrates superior attention capability compared to the state-of-the-art baseline models.

## Discussion on limitations

Despite the remarkable performance gains achieved by AMCANet in the task of few-shot skin lesion classification, the proposed model still exhibits several inherent limitations that warrant in-depth investigation. First, from the dataset perspective, although we have conducted comprehensive validation on the HAM10000 and PAD-UFES-20 datasets, both benchmarks are primarily collected from restricted populations and specific imaging devices. In comparison, the Derm104 dataset developed by Chen et al. [39] encompasses 104 distinct skin disease categories and incorporates a large volume of real-world clinical images, which covers far more diverse clinical scenarios. Accordingly, the generalization capability of our model in broader and more complex clinical settings remains to be further rigorously validated. Second, concerning the model architecture design, while the devised adaptive multi-scale convolution module enables dynamic receptive field adjustment to adapt to skin lesions of varying sizes, it may suffer from limitations in capturing long-range cross-scale dependencies, which hinders the full exploitation of multi-scale contextual information of skin lesions [18]. Furthermore, although the proposed skin spatial attention module leverages gradient information to enhance the extraction of critical boundary features of skin lesions, the joint integration of pixel-wise and channel-wise adaptive attention mechanisms is expected to further boost the model’s sensitivity to subtle edge details and textural features of lesions [40]. This feasible optimization strategy provides a promising direction for refining the AMCANet framework in future research. Finally, for clinical translation and practical deployment, the proposed model still requires thorough evaluation in real clinical workflows, including its deployment feasibility, real-time inference efficiency, and robustness against variable imaging conditions (e.g., uneven illumination, inconsistent shooting angles). These pivotal issues represent key research directions that need to be prioritized and addressed in subsequent studies.

## Conclusion

Our study addresses key challenges in classifying skin lesions, including the diversity of lesion scales, boundary ambiguity, intra-class morphological differences, and limited sample size. We propose a novel Adaptive Multi-Scale Convolutional Attention Network (AMCANet). By designing three dedicated modules: an adaptive multi-scale convolutional module, a hierarchical channel attention module, and a skin-specific spatial attention module, AMCANet achieves dynamic adaptation to lesion sizes, effective integration of multi-level semantic information, and enhancement of skin-specific boundary and texture features. The entire network maintains stable feature learning and classification performance, even with a minimal number of labeled samples, thereby addressing the issues of overfitting and generalization that arise from limited data availability. Extensive experiments on two public datasets, HAM10000 and PAD-UFES-20, demonstrate that AMCANet demonstrates promising results across various few-shot settings, with significant improvements in key metrics such as accuracy and F1-score compared to a range of advanced baseline models, including ConvNeXt, MuRANet, and BIIGMANet. Confusion matrix analysis reveals that AMCANet excels at distinguishing clinically confusing categories (such as MEL or NV) and maintains stable performance on heterogeneous images captured by smartphones (PAD-UFES-20 dataset). Generalization error analysis and feature space visualization further confirm that the features learned by AMCANet exhibit improved intra-class compactness and inter-class separability, accurately focusing on lesion areas, thus enhancing the model’s interpretability and clinical reliability. The theoretical value of this research lies in its groundbreaking combination of adaptive modulation, hierarchical semantic fusion, and boundary gradient enhancement, providing a new architecture for few-shot learning tailored for complex medical imaging. The proposed model offers a promising technological pathway and contributes to the foundational work for advancing automated auxiliary diagnosis of skin lesions.

## References

[1] Song Y, Zhang J, Liu Z, Xu Y, Quan S, Sun L, et al. Deep learning for hyperspectral image classification: a comprehensive review and future predictions. Inf Fusion. 2025;123:103285. doi: 10.1016/j.inffus.2025.103285

[2] Yang S, Du Y, Du S, Li X, Chen X, Li Y, et al. Few-shot cyberviolence intent classification with Meta-learning AutoEncoder based on adversarial domain adaptation. Neurocomputing. 2025;620:129089. doi: 10.1016/j.neucom.2024.129089

[3] Zhao J, Qin P, Mei Z, Zhao T. A multiple attentions-based multilevel hybrid-guided deep fuzzy convolutional neural network for image recognition. IEEE Trans Fuzzy Syst. 2025;33(8):2614–28.

[4] Sun X, Huang L, Xiao BG, Zhang Q, Li JQ, Ding YH, et al. X-ray computed tomography in metal additive manufacturing: a review on prevention, diagnostic, and prediction of failure. TWS. 2025;207:112736. doi: 10.1016/j.tws.2024.112736

[5] Sun Y, Wang L, Li G, Lin W, Wang L. A foundation model for enhancing magnetic resonance images and downstream segmentation, registration and diagnostic tasks. Nat Biomed Eng. 2025;9(4):521–38. doi: 10.1038/s41551-024-01283-7 39638876 PMC12360180

[6] Pacal I, Ozdemir B, Zeynalov J, Gasimov H, Pacal N. A novel CNN-ViT-based deep learning model for early skin cancer diagnosis. Biomed Signal Process Control. 2025;104:107627. doi: 10.1016/j.bspc.2025.107627

[7] Tan L, Wu H, Zhu J, Liang Y, Xia J. Clinical-inspired skin lesions recognition based on deep hair removal with multi-level feature fusion. Pattern Recognition. 2025;161:111325. doi: 10.1016/j.patcog.2024.111325

[8] Hu S, Zhang Z, Ying L, Lang G. Skin lesion classification with mini-batch sampling and deep metric learning. Applied Soft Computing. 2025;185:113850. doi: 10.1016/j.asoc.2025.113850

[9] Li Z, Jiang S, Xiang F, Li C, Li S, Gao T, et al. White patchy skin lesion classification using feature enhancement and interaction transformer module. Biomedical Signal Processing and Control. 2025;107:107819. doi: 10.1016/j.bspc.2025.107819

[10] Dakhli R, Barhoumi W. Improving skin lesion classification through saliency-guided loss functions. Comput Biol Med. 2025;192(Pt B):110299. doi: 10.1016/j.compbiomed.2025.110299 40375427

[11] Yang S, Du Y, Zheng X, Li X, Chen X, Li Y, et al. Few-shot intent detection with self-supervised pretraining and prototype-aware attention. Pattern Recognit. 2024;155:110641. doi: 10.1016/j.patcog.2024.110641

[12] Yang B, Zhang R, Peng H, Guo C, Luo X, Wang J, et al. SLP-Net:an efficient lightweight network for segmentation of skin lesions. Biomed Signal Process Control. 2025;101:107242. doi: 10.1016/j.bspc.2024.107242

[13] He F, Wu R, Zeng X, Song H, Li G, Wei Z. Skin lesion classification network based on improved MobileViT. Eng Appl Artif Intell. 2025;159:111726. doi: 10.1016/j.engappai.2025.111726

[14] Aruk I, Pacal I, Toprak AN. A novel hybrid ConvNeXt-based approach for enhanced skin lesion classification. Expert Syst Appl. 2025;283:127721. doi: 10.1016/j.eswa.2025.127721

[15] Gajera HK, Nayak DR, Zaveri MA. MTA-Net: multi-scale triplet attention-aware network for multiclass skin lesion classification. Comput Biol Med. 2025;196(Pt A):110729. doi: 10.1016/j.compbiomed.2025.110729 40652759

[16] Ghazouani H. Multi-residual attention network for skin lesion classification. Biomed Signal Process Control. 2025;103:107449. doi: 10.1016/j.bspc.2024.107449

[17] Tschandl P, Rosendahl C, Kittler H. The HAM10000 dataset, a large collection of multi-source dermatoscopic images of common pigmented skin lesions. Sci Data. 2018;5:180161. doi: 10.1038/sdata.2018.161 30106392 PMC6091241

[18] Ren Y, Xu W, Mao Y, Wu Y, Fu B, Thanh DNH. Few‐shot learning for dermatological conditions with Lesion Area Aware Swin Transformer. Int J Imaging Syst Technol. 2023;33(5):1549–60. doi: 10.1002/ima.22891

[19] Wang J, Chen F, Ma Y, Wang L, Fei Z, Shuai J, et al. XBound-former: toward cross-scale boundary modeling in transformers. IEEE Trans Med Imaging. 2023;42(6):1735–45. doi: 10.1109/TMI.2023.3236037 37018671

[20] Khurshid M, Singh R, Vatsa M. Multimodal dual-stage feature refinement for robust skin lesion classification. Sci Rep. 2025;15(1):37775. doi: 10.1038/s41598-025-14839-7 41162437 PMC12572157

[21] Noman A, Beiji Z, Zhu C, Alhabib M, Al-Sabri R. FEGGNN: Feature-Enhanced Gated Graph Neural Network for robust few-shot skin disease classification. Comput Biol Med. 2025;189:109902. doi: 10.1016/j.compbiomed.2025.109902 40056840

[22] Pacheco AGC, Lima GR, Salomão AS, Krohling B, Biral IP, de Angelo GG, et al. PAD-UFES-20: a skin lesion dataset composed of patient data and clinical images collected from smartphones. Data Brief. 2020;32:106221. doi: 10.1016/j.dib.2020.106221 32939378 PMC7479321

[23] Roy D, Dutta S, Bose S, Schwenker F, Sarkar R. Background-invariant independence-guided multi-head attention network for skin lesion classification. Proceedings of the Medical Image Computing and Computer Assisted Intervention; Cham; 2025. p. 34–44.

[24] Yu X, Xiong G, Wu J, Zheng J, Liang M, Qiu L, et al. Variational AdaBoost knowledge distillation for skin lesion classification in dermatology images. Complex Intell Syst. 2024;10(5):6787–804. doi: 10.1007/s40747-024-01501-4

[25] A RS, Chamola V, Hussain Z, Albalwy F, Hussain A. A novel end-to-end deep convolutional neural network based skin lesion classification framework. Expert Syst Appl. 2024;246:123056. doi: 10.1016/j.eswa.2023.123056

[26] Dzieniszewska A, Garbat P, Piramidowicz R. Deep pixel-wise supervision for skin lesion classification. Comput Biol Med. 2025;193:110352. doi: 10.1016/j.compbiomed.2025.110352 40398263

[27] Perez E, Kiela D, Cho K. True few-shot learning with language models. Proceedings of the 34th Annual Conference on Neural Information Processing Systems. Online; 2021. p. 11054–70.

[28] Snell J, Swersky K, Zemel RS. Prototypical networks for few-shot learning. Proceedings of the 30th Annual Conference on Neural Information Processing Systems; Long Beach, CA, USA; 2017. p. 4077–87.

[29] Finn C, Abbeel P, Levine S. Model-agnostic meta-learning for fast adaptation of deep networks. Proceedings of the 34th International Conference on Machine Learning. vol. 70; Sydney, NSW, Australia; 2017. p. 1126–35.

[30] Schick T, Schütze H. True few-shot learning with prompts—A real-world perspective. TACL. 2022;10:716–31.

[31] Winata GI, Madotto A, Lin Z, Liu R, Yosinski J, Fung P. Language models are few-shot multilingual learners. Proceedings of the 1st Workshop on Multilingual Representation Learning; Punta Cana, Dominican Republic; 2021. p. 1–15.

[32] Liu Z, Mao H, Wu C, Feichtenhofer C, Darrell T, Xie S. A ConvNet for the 2020s. Proceedings of IEEE/CVF Conference on Computer Vision and Pattern Recognition. New Orleans, LA, USA; 2022. p. 11966–76.

[33] Hosny KM, Said W, Elmezain M, Kassem MA. Explainable deep inherent learning for multi-classes skin lesion classification. Appl Soft Comput. 2024;159:111624. doi: 10.1016/j.asoc.2024.111624

[34] Paszke A, Gross S, Massa F, Lerer A, Bradbury J, Chanan G, et al. Pytorch: an imperative style, high-performance deep learning library. Adv Neural Inf Process Syst. 2019;32.

[35] Kingma DP, Ba J. Adam: a method for stochastic optimization. In: Bengio Y, LeCun Y, editors. Proceedings of the 3rd International Conference on Learning Representations; San Diego, CA, USA; 2015.

[36] He Y-L, Chen C-J, Chen J-Q. A data-driven fused kernel density estimator for multi-modal distribution. Inf Fusion. 2026;125:103434. doi: 10.1016/j.inffus.2025.103434

[37] van der Maaten L, Hinton G. Visualizing data using t-SNE. J Mach Learn Res. 2008;9(86):2579–605.

[38] Selvaraju RR, Cogswell M, Das A, Vedantam R, Parikh D, Batra D. Grad-CAM: visual explanations from deep networks via gradient-based localization. Proceedings of IEEE International Conference on Computer Vision; Venice, Italy; 2017. p. 618–26.

[39] Chen T, Liu Q, Yang J. Few-shot classification with multiscale feature fusion for clinical skin disease diagnosis. Clin Cosmet Investig Dermatol. 2024;17:1007–26. doi: 10.2147/CCID.S458255 38737944 PMC11088239

[40] Zafar A, Aftab D, Qureshi R, Fan X, Chen P, Wu J, et al. Single stage adaptive multi-attention network for image restoration. IEEE Trans Image Process. 2024;33:2924–35. doi: 10.1109/TIP.2024.3384838 38598372

